# Metformin enhances the antitumor activity of oncolytic herpes simplex virus HF10 (canerpaturev) in a pancreatic cell cancer subcutaneous model

**DOI:** 10.1038/s41598-022-25065-w

**Published:** 2022-12-13

**Authors:** Mohamed Abdelmoneim, Ibrahim Ragab Eissa, Mona Alhussein Aboalela, Yoshinori Naoe, Shigeru Matsumura, Patricia Angela Sibal, Itzel Bustos-Villalobos, Maki Tanaka, Yasuhiro Kodera, Hideki Kasuya

**Affiliations:** 1 Graduate School of Medicine, Cancer Immune Therapy Research Center, Nagoya University, Nagoya, Japan; 2 Department of Surgery II, Graduate School of Medicine, Nagoya University, Nagoya, Japan; 3 Department of Microbiology, Faculty of Veterinary Medicine, Zagazig University, Zagazig, Egypt; 4 Faculty of Science, Tanta University, Tanta, Egypt; 5 Medical Microbiology and Immunology Department, Faculty of Medicine, Zagazig University, Zagazig, Egypt; 6 Takara Bio Inc., Kusatsu, Shiga Japan

**Keywords:** Cancer immunotherapy, Tumour immunology

## Abstract

Oncolytic virus (OV) therapy is a promising cancer immunotherapy, especially for cold tumors by inducing the direct lysis of cancer cells and initiation of potent antitumor response. Canerpaturev (C-REV) is an attenuated oncolytic herpes simplex virus-1, which demonstrated a potent antitumor effect in various preclinical models when used either alone or combined. Metformin is a commonly prescribed antidiabetic drug that demonstrated a potent immune modulator effect and antitumor response. We combined C-REV with metformin in a low immunogenic bilateral murine tumor model to enhance C-REV’s antitumor efficacy. In vitro, metformin does not enhance the C-REV cell cytotoxic effect. However, in in vivo model, intratumoral administration of C-REV with the systemic administration of metformin led to synergistic antitumor effect on both sides of tumor and prolonged survival. Moreover, combination therapy increased the effector CD44^+^ CD8^+^ PD1^-^ subset and decreased the proportion of terminally-differentiated CD103^+^ KLRG-1^+^ T-regulatory cells on both sides of tumor. Interestingly, combination therapy efficiently modulates conventional dendritic cells type-1 (cDC1) on tumors, and tumor-drained lymph nodes. Our findings suggest that combination of C-REV and metformin enhances systemic antitumor immunity. This study may provide insights into the mechanism of action of OV therapy plus metformin combination against various tumor models.

## Introduction

Pancreatic ductal adenocarcinoma (PDAC) is the most common form of pancreatic cancer and it represents the fourth leading cause of cancer deaths in Japan, with forecasts indicating a further escalation of mortality rates in the coming decades^1^. Now, the current standard of care for patients with PDAC focuses on chemotherapeutic regimens and pancreatic cancer surgery. However, limited treatment options, advanced tumor stages due to late diagnosis, and the aggressive behavior of PDAC contribute to the high mortality of the disease^2^. Consequently, there is an urgent need for an alternative approach to pancreatic cancer treatment. Recently, immunotherapy has been considered a promising approach to cancer treatment. It has a potential impact on the treatment of different types of cancers with mild to moderate side effects on patients^3^. Even though advancements in cancer immunotherapy, it showed limited preclinical and clinical response against pancreatic cancer^4^. In addition to this, PDAC patients showed no response to a single treatment by immune checkpoint inhibitors (ICIs)^5^. Additionally, combination ICIs with chemotherapy have not either induced marked effects in patients^4^. The limitations of treatments are attributed to its cold tumor microenvironment (TME) with low MHC-I expression^6^. These are accompanied by high infiltration of suppressive immune cells, as well as the physical limitations like the presence of fibrotic tissue and stellate cells^7,8^. These factors impair cytotoxic CD8^+^ T cells infiltration and lead to T cell exhaustion^9^. Therefore, to improve the outcomes, other approaches are critically needed to beat PDAC resistance^10^.

One of the immune therapeutic targeted agents that has the potential against cold tumors is Oncolytic viruses (OVs). OV therapy is a promising alternative for patients who does not respond to ICIs due to their dual benefit in one therapy^11^. OVs are attenuated viruses that cause anticancer effects by directly killing cancer cells (oncolysis) as well as promoting antitumor immunity. Most OVs infect tumor cells through virus-specific receptors, which allows the virus to replicate in the tumor regardless of MHC-I expression status. OVs can convert a cold tumor into a hot tumor by increasing the infiltration of immune cells upon virus infection accompanied by pathogen-associated molecular patterns (PAMPs) and danger-associated molecular patterns (DAMPs)^12^.

Canerpaturev (C-REV) is a promising OV, which was originally isolated from herpes simplex virus-1 (HSV-1) strain HF as clone 10 (Previously known as HF10). C-REV has a unique dsDNA genomic structure with non-engineered two deletions (3832 bp were deleted at the *UL56*/IRL junction and 2295 bp were deleted at the TRL region) and without any foreign gene’s insertion^13,14^. These are accompanied by genetic arrangement and frame-shift mutations that led to loss expression of *UL43*, *UL49.5*, *UL55*, and latency-associated transcript (LAT) genes, which attenuates its pathogenicity and neuroinvasiveness^15^. C-REV showed potent antitumor effects against various preclinical models, including pancreatic cancer^14^. C-REV combined with anti-PD-L1 showed a greater antitumor effect with high infiltration of CD8^+^ PD-1^-^ tumor-infiltrating lymphocyte cells (TILs) in SCC-VII model^16^. C-REV combined with chemotherapeutic S-1 also enhanced antitumor efficacy in a murine triple-negative breast cancer model^17^. It also demonstrated its safety and efficacy in phases I, and II clinical trials targeting melanoma, pancreatic, breast, head, and neck cancer^14^. C-REV combined with gemcitabine and nab-paclitaxel in phase I clinical trial against unresectable stage III or IV pancreatic cancer, and showed a favorable benefit/risk profile with antitumor activity^18^. Furthermore, C-REV combined with cetuximab and bevacizumab synergistically inhibited the growth of human colorectal cancer as well as human breast carcinoma xenograft, respectively^19,20^. Concluding with those results that the combination approach is promising and highlighting the need of new combination therapy to overcome cold tumors.

Metformin is an FDA-approved, commonly prescribed systemic antidiabetic medication for type 2 diabetes patients. Recently, metformin demonstrated its cancer antitumor effectiveness. Several epidemiological studies showed that metformin reduces cancer incidence in type 2 diabetes patients and improves their prognosis^21^. Metformin improved the survival of diabetic patients with pancreatic cancer^22^. Metformin affects the growth of cancer cells, which depend on mitochondrial bioenergetics through inhibition of the complex I of the electron transport chain (ETC). ETC inhibition leads to the accumulation of NADH inside the cells, which negatively affects ATP production. Diminished ATP levels in the cell leads to AMP-activated protein kinase (AMPK) activation, which inhibits the mammalian target of rapamycin (mTOR), thus inhibiting cell growth^23^. In addition to direct inhibition of tumor cells, metformin showed a potent immune modulator effect^24^. Metformin targets CD8^+^ cells in the TME; it maintains high cytotoxic T lymphocyte (CTL) activity in tumor tissues^25^. Moreover, metformin modulates suppressive immune cells in the TME.^24^ It has a negative impact on induced T-regulatory generation and tumor-infiltrating T regulatory (Ti-Treg) cells^26^. It also inhibits the suppressive function of CD11b^+^ myeloid subsets, especially myeloid-derived suppressive cells (MDSCs) and tumor-associated macrophages^27,28^. Additionally, metformin suppresses the induction of M2-like polarization of macrophages in tumor-bearing mice^29^. Consequently, metformin mainly modulates immune suppressive cells, which could be ideal for combining with OVs against cold tumors such as pancreatic cancer.

In this paper, we hypothesized that the combination of C-REV as an oncolytic virus plus metformin might be a good option for pancreatic cancer treatment. This is the first paper to show the effect of OVs combined with metformin on the TME. This study used a low immunogenic Pan02 murine PDAC model due to its high morbidity and mortality^2^. We combined the oncolytic virus C-REV with the antidiabetic drug metformin; the combination induced a significant antitumor immunity in comparison to single treatments in bilateral tumor-bearing mice. The combination treatment increased the effector CD44^+^ CD8^+^ PD1^−^ subset and decreased the proportion of terminally-differentiated CD103^+^ KLRG-1^+^ T-regulatory cells, as well as increased XCR-1 expression on conventional dendritic cells type-1 (cDC1) in the tumor, and tumor-drained lymph nodes (TDLNs). These results may provide insights into the mechanism of this combination therapy against low immunogenic tumors.

## Results

### Metformin promotes the antitumor activity of C-REV on both the injected and contralateral sides in Pan02 tumor-bearing mice

**Figure 1:**
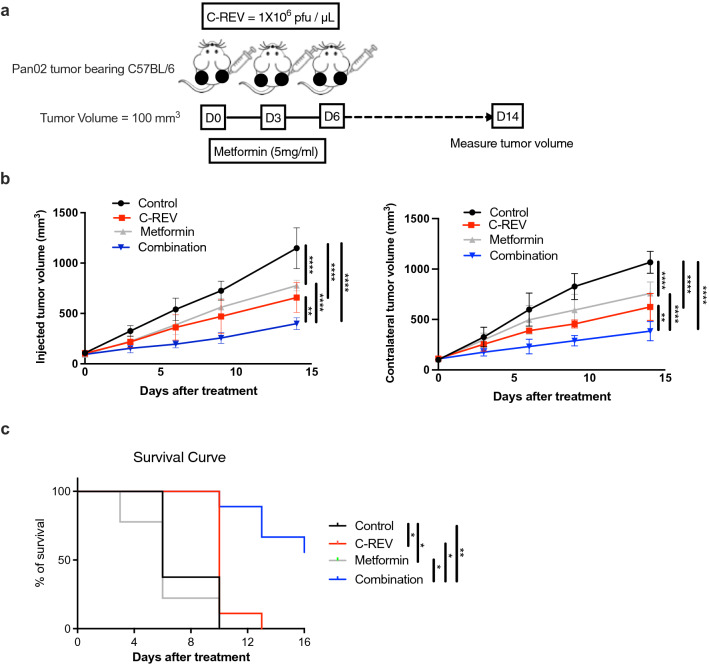
Metformin promotes antitumor activity of C-REV on both the injected and contralateral sides in Pan02 tumor-bearing mice. (**a**) The scheme shows the schedule of C-REV and metformin treatment in C57BL/6 bilateral tumor-bearing mice. Female Six- to seven-week-old C57BL/6 mice were subcutaneously inoculated with Pan02 in both flanks. When tumor size reached around 100 mm^3^, mice were randomly divided into four groups (n = 4 mice) with an equal average tumor volume among the groups. C-REV was injected three times (1 × 10^6^  pfu) with three days intervals (D0, D3, and D6) on only one side (injected side) and metformin was continuously supplied in the drinking water (5 mg/ml) after C-REV treatment. Tumor sizes on both sides of tumor were measured twice a week. This experiment was conducted four times, yielding similar results. (**b**) Representative tumor growth in Pan02 tumor models after treatment. Data are presented as mean ± SD. Two-way ANOVA followed by Tukey’s multiple comparisons tests were performed. (**c**) Representative mouse survival in Pan02 tumor models after treatment (n = 9). For the evaluation of survival, the death event was defined when the total tumor size reached 2000 mm^3^. Survival analysis was performed using the Kaplan–Meier method. The log-rank test was used for the statistical comparison of the curves. (**b**) and (**c**) are from different experiments. * p < 0.05, ** p < 0.01, **** p < 0.0001.

The PD-1/PD-L1 interaction decreases CD8^+^ T cells activity. We previously reported that PD-L1 is highly expressed on Pan02 and IFNs treatment significantly enhanced its expression^16^. Pan02 tumors are resistant to PD-1 blockade therapy, and therefore, they are characterized as unresponsive tumors with low sensitivity to acquired immunity. The immune ignorance of Pan02 is attributed to a lack of MHC-I expression^30^. Thus, new approaches are needed to overcome Pan02 cold tumor resistance. C-REV has a powerful antitumor effect even in PD-L1-enriched tumors^16^. Metformin recently showed a potent immune modulatory effect. Therefore, we evaluated whether the combined effect of C-REV with metformin could enhance antitumor activity using the bilateral Pan02 tumor model. Mice were subcutaneously inoculated on both flanks. When tumor size reached around 100 mm^3^, C-REV was injected three times (1 × 10^6^ pfu) with three days intervals (D0, D3, and D6) on only one side (referred to as the injected side; the non-injected side is the contralateral side) and metformin continuously supplied in the drinking water (5 mg/ml) after C-REV treatment (Fig. 1a). Tumor sizes on both sides were measured twice a week. C-REV and metformin as a single treatment showed significant antitumor activity against the control group on both the injected and contralateral sides. However, combination therapy enhanced significant antitumor activity in comparison to C-REV and metformin monotherapies on both the injected and contralateral sides (Fig. 1b). In the metformin group, the size of injected and contralateral tumors was almost comparable due to the systemic presence of metformin on both sides. Although C-REV was injected on one side in the combination group, there was a significant antitumor effect on the contralateral side in comparison to single treatments groups (Fig. 1b). Moreover, C-REV and metformin as single treatment prolonged survival in vivo compared to the control group. In addition to that, the combination therapy prolonged survival compared to single-treated groups (Fig. 1c). Interestingly, combination therapy showed synergism from the calculation of the synergistic effect on both the injected and contralateral sides (Table 1). Overall, our data suggested that the onco-suppressive activity of C-REV could be enhanced by metformin against a PD-1-resistance Pan02 tumor.

**Table 1:** Fractional tumor volume (FTV) after treatment with C-REV, either alone or in combination with metformin.

Injected side						Contralateral side					
	FTV	FTV	E-FTV	O-FTV	E-FTV/O-FTV^a^		FTV	FTV	E-FTV	O-FTV	E-FTV/O-FTV^a^
Days	C-REV	Met	Comb	Comb	Comb	Days	C-REV	Met	Comb	Comb	Comb
0	0.97103129	0.88412514	0.85851318	0.86906141	0.9878625	0	1.1159601	1.0286783	1.14796394	1.09600998	1.04740282
3	0.67217525	0.68985396	0.46370276	0.47386626	0.97855197	3	0.78076923	0.91846154	0.71710651	0.54192308	**1.32326254**
6	0.66928952	0.71765795	0.48032094	0.36241611	**1.32533001**	6	0.65195566	0.83078854	0.54163729	0.38883079	**1.39298971**
9	0.6487931	0.77431034	0.50236721	0.35506897	**1.41484405**	9	0.55093712	0.71659613	0.39479941	0.35	**1.12799831**
14	0.5735134	0.67730342	0.38844258	0.34741886	**1.11808144**	14	0.5846316	0.71078833	0.41554932	0.36066534	**1.15217427**

FTV, fractional tumor volume (mean tumor volume experimental/mean tumor volume control); E- FTV, expected FTV (mean FTV of C-REV) × (mean FTV of metformin); O-FTV, observed FTV.

^a^Synergic effect: E-FTV/O-TV>1 in bold.

### Metformin does not enhance cell cytotoxicity induced by C-REV in vitro

C-REV plus metformin combination induced significant bilateral tumor regression and prolonged survival. Metformin modulates the cell cytotoxicity depending on the concentration of metformin in the medium as well as the cellular glycemic status^31^. To examine this, we checked different low concentrations of metformin (10 μM, 100 μM and 1000 μM) on pan02 cell line using two different glucose conditions; high glucose (25 mM) and low glucose (5.5 mM) conditions in vitro and measure cytotoxicity by using MTT assay. Low concentrations of metformin did not statistically enhance cell cytotoxicity either in high or low glucose medium (Supplementary Fig. S1a).

Then, we examined the effect of glucose concentration on C-REV cell cytotoxicity in vitro. Our previous result revealed that C-REV induced cell cytotoxicity in a MOI- dependent manner in Pan02 cells cultured with high glucose medium^16^. The same results were observed in low glucose medium. However, no significant differences were detected between low and high glucose condition medium at the same MOI (Supplementary Fig. S1b). This indicates that both glucose conditions do not affect viral cell cytotoxicity.

To examine the rationale for using metformin in combination with C-REV; we compared the effect of C-REV and metformin combination using both glucose conditions. C-REV was infected into Pan02 with different MOI (0.1, 1, and 10) combined with different concentrations of metformin. On both glucose conditions, the metformin treatment did not enhance C-REV cell cytotoxicity (Supplementary Fig. S1c, d). These effects were sustained until day 3 after combination treatment in high glucose medium (Supplementary Fig. S1e).

Overall, these results suggest that micromolar concentrations of metformin did not significantly affect cell cytotoxicity either alone or in combination with C-REV.

To determine whether metformin treatment affects CREV replication in tumor cells, we titered C-REV from infected Pan02 cells to assess viral replication. We infected Pan02 with C-REV (MOI 1), and we incubated them with 100 μM of metformin for 2 days. Metformin had no effect on C-REV replication in Pan02 cell line (Supplementary Fig. S1f). Therefore, a low concentration of metformin did not interfere with C-REV replication in tumor cells.

### Combination therapy enhances infiltration and IFN-γ production from CD8^+^ CD3^+^ TILs

**Figure 2:**
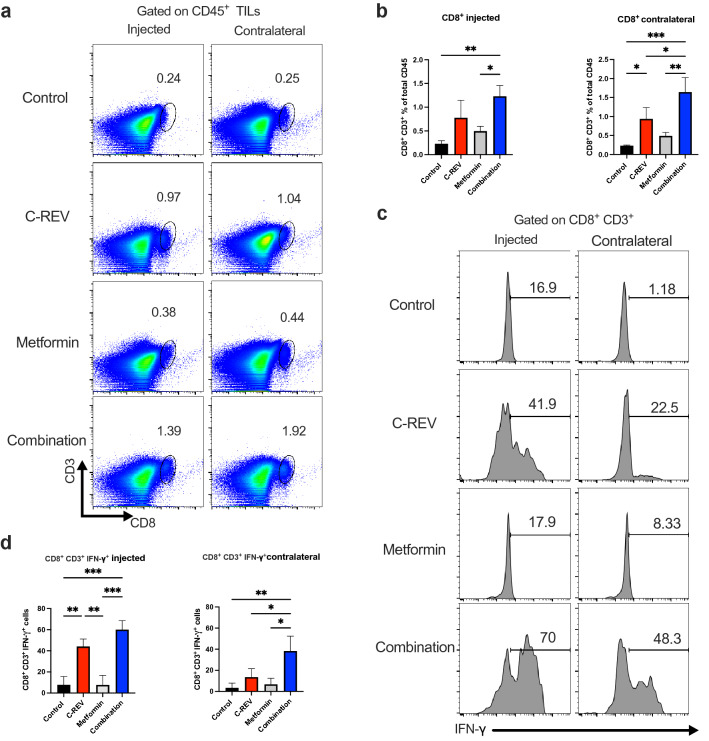
Combination therapy enhances infiltration and IFN-γ production from CD8^+^ CD3^+^ TILs. Mice were inoculated with Pan02 tumors as indicated in Fig. 1a, on day 14 after combination treatment, tumors from both injected and contralateral sides were harvested, dispersed into single cells by enzyme digestion, and stained with the indicated antibodies. Percentages of each population in each group are displayed in bar graphs. (**a**) Dot charts show CD8^+^ CD3^+^ T cells from Pan02 tumors on the injected and contralateral sides. (**b**) Bar graphs show the percentage of CD8^+^ CD3^+^ cells on the injected and contralateral sides. (**c**) Mice were inoculated with Pan02 tumors as in Fig. 1a, on day 14 after combination treatment, tumors and TDLNs were harvested and CD8^+^ CD3^+^ TILs were isolated by MACs and subjected them to anti-CD3/anti-CD28 antibody stimulation for 2 days. Histograms show representative intracellular IFN-γ production from CD8^+^ CD3^+^ TILs cells on the injected and contralateral sides. (**d**) Bar graphs show the percentage of IFN-γ production from CD8^+^ CD3^+^ TILs cells on the injected and contralateral sides. Data are presented as mean ± SD (n = 3 mice). One-way ANOVA with a post-hoc Tukey’s tests was performed. * p < 0.05, ** p < 0.01, *** p < 0.001.

In vitro, the combination of C-REV and metformin did not show a significant cytotoxic effect against Pan02. However, in vivo results showed that combination treatment had a significant antitumor effect on the contralateral side. To understand the mechanism of action of the rational combination between C-REV and metformin, we investigated the infiltration of immune cells into the tumor. As known, CD8^+^ T cells play important roles in the antitumor activity of C-REV^32^. Hence, we investigated the infiltration of CD8^+^ CD3^+^ TILs (Supplementary Fig. S2a) into the tumor using the schedule shown in Fig. 1a. Our result revealed that C-REV and metformin combination therapy significantly increased the percentage of CD8^+^ CD3^+^ TILs cells on both the injected and contralateral sides (Fig. 2a, b). Although C-REV was injected on one side, the percentage of CD8^+^ CD3^+^ TILs on the contralateral side was significantly increased in the combination group in comparison to single treatment groups. Recently, CD4^+^ T cells have shown multiple roles in antitumor immunity^33^. Therefore, we checked the CD4^+^ CD3^+^ T cells infiltration to examine whether our therapy mainly modulates this population. We found that combination treatment did not significantly increase CD4^+^ CD3^+^ TILs in the injected side (Supplementary Fig. S2b). These results indicated that combination therapy mainly modulates CD8^+^ CD3^+^ TILs, which may have a role in the antitumor activity of our combination therapy.

Next, we compared the functional activity of tumor-infiltrating CD8^+^ CD3^+^ T cells after single and combination treatment by measuring IFN-γ production. Here, we investigated two different treatment schemes to monitor IFN-γ production. First, C-REV was injected one time, and metformin was daily supplied in the drinking water. CD8^+^ CD3^+^ TILs were isolated on day 3 after treatment and subjected them to anti-CD3/anti-CD28 antibody stimulation for 2 days (Supplementary Fig. S2c). IFN-γ production did not show a significant difference between the control and the treated groups (Supplementary Fig. S2d, e). This result suggested that three days schedule was not enough to assess the functional state of CD8^+^ TILs and intratumoral injection of C-REV might induce over-stimulation of pre-existed CD8^+^ CD3^+^ TILs which enhance them to apoptosis. Then, we used the main scheme (Supplementary Fig. S2f). On day 14 both tumor sides and both injected and contralateral TDLNs (axillary and inguinal lymph nodes) were collected then CD8^+^ CD3^+^ T cells were isolated and stimulated for 2 days. Stimulated CD8^+^ CD3^+^ T cells from injected side of C-REV and combination groups showed a significant increase in IFN-γ production against the control group. Furthermore, enhanced IFN-γ production was observed in the stimulated cells derived from contralateral tumors of the combination group compared to single-treated groups (Fig. 2c, d). However, no significant differences were detected from CD8^+^ CD3^+^ T cells isolated from TDLNs either after single or combination treatment (Supplementary Fig. S2g). Therefore, the significant infiltration of CD8^+^ CD3^+^ T cells, which were detected on the contralateral side of the combination group was accompanied by significant IFN-γ production. These data demonstrated that combination treatment induces infiltration of CD8^+^ CD3^+^ T cells into tumors with high IFN-γ production on both the injected and contralateral sides. Together, we confirm that metformin enhances C-REV antitumor activity by targeting immune cells infiltrated into the tumor.

### Combination therapy promotes effector CD44^+^ CD8^+^ PD1^−^ and decreased proportion of terminally-differentiated CD103^+^ KLRG-1^+^ T-regulatory cells

C-REV treatment induced the infiltration of CD8^+^ PD-1^−^ TILs in SCC-VII murine tumor model^16^. In Pan02 murine tumor model, we also detected that C-REV single treatment significantly enhanced CD8^+^ PD-1^−^ TILs on both sides of tumor in comparison to the control group. In the metformin group, CD8^+^ PD-1^−^ TILs were increased compared to the control group, especially on the contralateral side. Interestingly, the combination of C-REV and metformin enhanced more infiltration of CD8^+^ PD-1^−^ on the injected and contralateral sides compared to single-treated groups (Supplementary Fig. S3a, b). Additionally, we checked CD4^+^ PD-1^−^ TILs infiltration on both sides of tumor. CD4^+^ PD-1^−^ TILs significantly increased on both sides of tumor after treatment with either C-REV alone or combined (Supplementary Fig. S3c). Also, we detected significant changes in CD8^+^ PD-1^−^ TILs among treated groups. Therefore, combination treatment mainly modulates CD8^+^ PD-1^−^ TILs.

**Figure 3:**
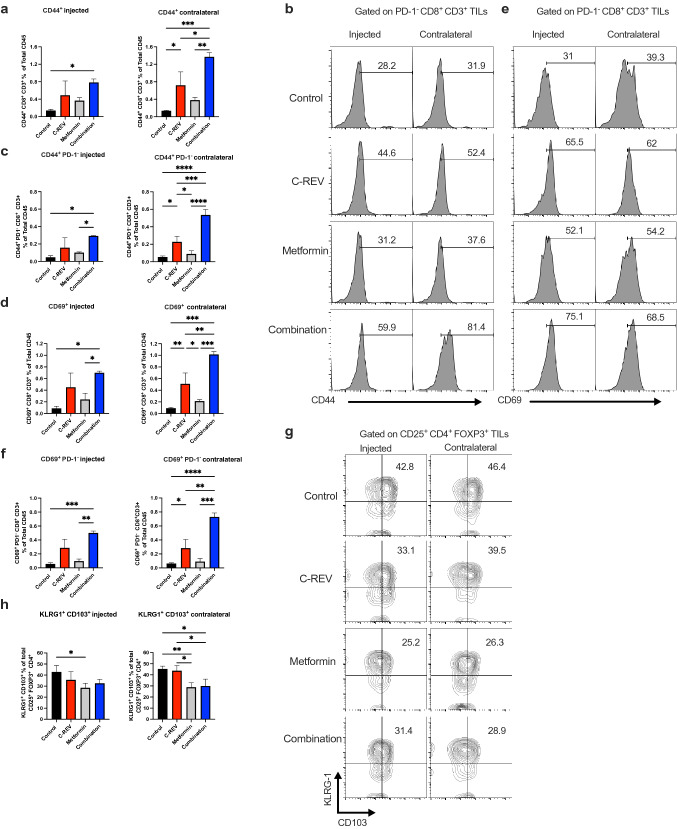
Combination therapy promotes effector CD44^+^ CD8^+^ PD1^-^ and decreased terminally-differentiated CD103^+^ KLRG-1^+^ T-regulatory cells. Mice were inoculated with Pan02 tumors as indicated in Fig. 1a, and tumors and TDLNs were collected as previously shown. (**a**) Bar graphs show the percentage of CD44^+^ CD8^+^ CD3^+^ cells on the injected and contralateral sides. (**b**) Representative histograms show CD44^+^ expression on CD8^+^ CD3^+^ PD-1^−^ cells on the injected and contralateral sides. (**c**) Bar graphs show the percentage of CD44^+^ PD-1^−^ CD8^+^ CD3^+^ cells on the injected and contralateral sides. (**d**) Bar graphs show the percentage of CD69^+^ CD8^+^ CD3^+^ cells on the injected and contralateral sides. (**e**) Representative histograms show CD69^+^ expression on CD8^+^ CD3^+^ PD-1^−^ cells on the injected and contralateral sides. (**f**) Bar graphs show the percentage of CD69^+^ PD-1^−^ CD8^+^ CD3^+^ cells on the injected and contralateral sides. (**g**) A representative dot plot shows CD103^+^ KLRG-1^+^ expression on CD4^+^ CD25^+^ FOXP3^+^ tumor-infiltrating T-reg cells on the injected and contralateral sides. (**h**) Bar graphs show the percentage of CD103^+^ KLRG-1^+^ expression on CD4^+^ CD25^+^ FOXP3^+^ tumor-infiltrating T-reg cells on the injected and contralateral sides. Data are presented as mean ± SD (n = 4 mice). This experiment was conducted at least two times. One-way ANOVA with a post-hoc Tukey’s tests was performed. * p < 0.05, ** p < 0.01, *** p < 0.001, **** p < 0.0001.

CD8^+^ CD44^+^ and CD8^+^ CD69^+^ TILs are activated effector cells with an antitumor immune response^34,35^. Thus, we checked CD44^+^ expression as an effector marker on CD8^+^ CD3^+^ TILs. Then, we checked its expression on CD8^+^ PD-1^−^ TILs (Supplementary Fig. S3d). Combination treatment significantly increased CD44^+^ CD8^+^ infiltration accompanied by a significant increase in CD44^+^ CD8^+^ PD-1^−^ population on both the injected and contralateral sides (Fig. 3a–c). Additionally, we checked the expression of CD69^+^ as an activation marker on CD8^+^ CD3^+^ TILs as well as its expression on CD8^+^ PD-1^−^ TILs. Our results showed that combination treatment significantly induced CD69^+^ CD8^+^ infiltration with a significant increase in CD69^+^ CD8^+^ PD-1^−^ population on both sides of tumor (Fig. 3d–f). On the contralateral side of the combination group, there were a significant increase in CD44^+^ and CD69^+^ CD8^+^ as well as CD44^+^ and CD69^+^ CD8^+^ PD-1^−^ populations in comparison to single-treated groups, which indicates antitumor specificity. These findings elucidate that, combination therapy efficiently enhances infiltration of both CD44^+^ and CD69^+^ CD8^+^ PD1^-^ TILs, which may have an antitumor effect. Furthermore, we examined CD44^+^ and CD69^+^ expression in CD8^+^ CD3^+^ TDLNs (Supplementary Fig. S3e). No changes were detected in effector CD44^+^ CD8^+^ or CD69^+^ CD8^+^ in TDLNs (Supplementary Fig. S3f).

Next, we examined CD4^+^ CD25^+^ FOXP3^+^ Ti-Treg cells. The combination treatment did not affect the percentage of infiltrated CD25^+^ FOXP3^+^ CD4^+^ cells on both sides (Supplementary Fig. S3g). However, we found that both metformin and combination treatment have the tendency to decrease the proportion of terminally-differentiated CD103^+^ KLRG-1^+^ cells from Ti-Treg cells, especially on the contralateral side (Fig. 3g, h). To clarify the antitumor effect of combination treatment, we calculated the ratio between effector CD44^+^ CD8^+^ PD-1^−^ population and terminally differentiated CD103^+^ KLRG-1^+^ T-reg cells. Combination treatment significantly increased the ratio on both sides of tumor (Supplementary Fig. S3h). These results suggested that combination treatment induced an increase in the effector CD8^+^ TILs accompanied by a decrease in terminally-differentiated T-regulatory cells, which may contribute to superior antitumor immunity.

### Combination therapy efficiently enhances dendritic cell activity

**Figure 4:**
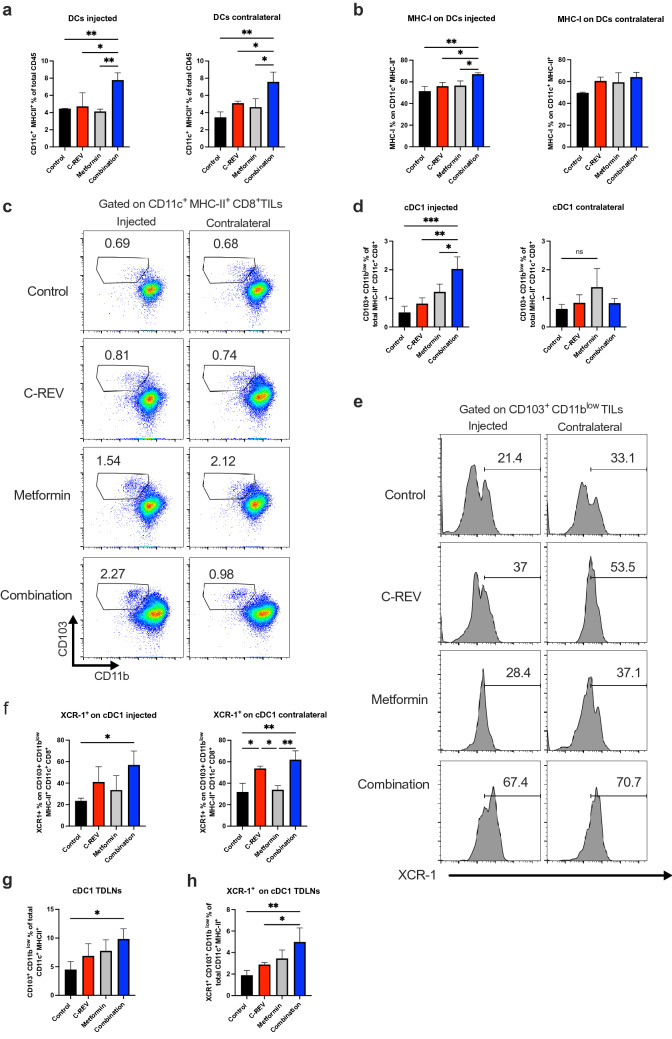
Combination therapy efficiently enhances dendritic cell activity. Mice were inoculated with Pan02 tumors as indicated in Fig. 1a, and tumors and TDLNs were collected as previously shown. (**a**) Bar graphs show the percentage of DCs (MHC-II^+^ CD11c^+^) on the injected and contralateral sides. (**b**) Bar graphs show the percentage of MHC-I expression on DCs on the injected and contralateral sides. (**c**) A representative dot plot shows cDC1 (CD103^+^ CD11b^low^) from cDCs (MHC-II^+^ CD11c^+^ CD8α^+^) on the injected and contralateral sides. (**d**) Bar graphs show the percentage of cDC1 from cDCs on the injected and contralateral sides. (**e**) Representative histograms show XCR-1 expression on cDC1 on the injected and contralateral sides. (**f**) Bar graphs show the percentage of XCR-1 expression on cDC1 on the injected and contralateral sides. (**g**) A bar graph shows the percentage of cDC1 from DCs in TDLNs. (**h**) A bar graph shows the percentage of XCR-1 expression on cDC1 in TDLNs. Data are presented as mean ± SD (n = 4 mice). This experiment was conducted at least two times. One-way ANOVA with a post-hoc Tukey’s tests was performed. * p < 0.05, ** p < 0.01, *** p < 0.001.

C-REV and metformin combination enhanced adaptive immune response by affecting both effector CD8^+^ PD-1^−^ TILs and terminally-differentiated Ti-Treg cells. Next, we assessed the effect of the combination group on innate dendritic cells (DCs). We evaluated the effect of combination treatment on the activation status of DCs on both TME and TDLNs (Supplementary Fig. S3i). Combination treatment significantly increased tumor-infiltrating DCs (CD11c^+^ MHC-II^+^) on both the injected and contralateral sides in comparison to single-treated groups (Fig. 4a). We also observed an increase in MHC-I on the infiltrated DCs, especially on the injected side (Fig. 4b). As known, cDC1 subset is essential for antitumor immunity as it plays a critical role in attraction, proper activation and maintenance of the effector function of CD8^+^ TILs^36,37^. Therefore, we checked the effect of combination treatment on the cDC1 population. Combination treatment significantly increased cDC1 (CD103^+^ CD11b^low^) percentage from total cDCs (CD11c^+^ MHC-II^+^ CD8α^+^) on the injected side compared to single-treated groups (Fig. 4c, d). Furthermore, we checked the expression of chemokine receptor XCR-1 on cDC1 as a marker for efficient cross-presenting DCs in the TME^38–40^. We found that combination treatment significantly enhanced XCR-1 expression on cDC1 on both the injected and contralateral sides (Fig. 4e, f).

Moreover, we examined the cDC1 population in the TDLNs. Combination treatment significantly increased the cDC1 population, which is essential for CD8^+^ T cell activation that infiltrated into the tumors after activation (Fig. 4g). We also detected a significant increase in XCR-1 expression on the cDC1 population in the TDLNs, suggesting that combination treatment enhanced the infiltration of the cross-presenting DCs toward TDLNs (Fig. 4h). Together, we concluded that our combination modulates the activity of innate DCs on both sides of tumor and TDLNs. It upregulates the infiltration of DCs and cDC1 populations, which may contribute to the antitumor activity.

## Discussion

In this study, we demonstrated the rational combination between oncolytic virus C-REV and metformin against a low immunogenic bilateral Pan02 tumor-bearing mice model. We propose a possibility for the combination treatment to enhance antitumor immunity. We showed that combination treatment significantly enhanced CD8^+^ TILs infiltration on both C-REV treated tumor and C-REV non-treated tumor, which play an essential role in antitumor activity. CD8^+^ TILs infiltration was accompanied by high levels of IFN-γ production, which indicates an activating status. The combination therapy also enhanced the high infiltration of effector CD44^+^ and CD69^+^ CD8^+^ PD-1^−^ on both sides of tumor, which may have a role in the antitumor response. Furthermore, the combination therapy reduced the proportion of terminally-differentiated CD103^+^ KLRG-1^+^ Ti-Treg cells in the tumors, improving the antitumor effect. In addition to that, the combination treatment also ameliorated the innate immune cells activity by increasing DCs infiltration and XCR-1 expression on cDC1, which helps CD8^+^ TILs activation.

As known, the efficacy of the oncolytic virus is highly based on its replication efficiency in tumor cells^41^. C-REV intratumoral injection induces strong tumor regression in different murine tumor models, which indicates its ability to replicate in low-nutrient TME^16,17,19^. Metformin also can reduce tumor growth in different murine models^25–28^. In murine models, oral administration of metformin reached a low concentration (micromolar range) in the tumor tissue^42^. In vitro, high concentrations of metformin as well as low glucose conditions are essential to induce cell cytotoxic effect against several cancer cell lines^31,43^. Since, the high concentrations of metformin could not be achieved in patients, here we examined low concentrations of metformin against Pan02 cells. Low concentrations of metformin can suppress murine tumor growth in vivo, while in vitro it could not suppress the growth of different cancer cell lines. Moreover, viral replication assays indicated that low concentration of metformin did not interfere with C-REV replication in tumor cells (Supplementary Fig. S1f). Therefore, we concluded that the synergistic effect of metformin and C-REV might be contributed to their effect on infiltrated immune cells.

The activity of CD8^+^ T cells plays an important role in the antitumor activity of C-REV^32^. C-REV increases PAMPs and DAMPs, which are phagocytosed by APCs, and activate antitumor CD8^+^ T cells^12^. Importantly, combination treatment enhances the high infiltration of CD8^+^ TILs with high IFN-γ production on the contralateral side, indicating the specificity and activity of infiltrated CD8^+^ TILs against tumors (Fig. 3a–d). It was reported that intratumoral injection of C-REV induces high CD8^+^ infiltration and IFN- γ production, which are specific against tumor antigens as clearance of HSV-1 genome from tumor after 1 week of virus injection was reported^44^. In MethA tumor-bearing mice and ovarian cancer murine model, metformin increases IFN-γ production from CD8^+^ TILs by enhancing glycolysis^45,46^. Moreover, the antitumor effect of metformin was completely abrogated in leukemia murine model after deletion of CD8^+^ T cells, implying the antitumor effect of CD8^+^ TILs cells^25^. In this study, the injected side of C-REV demonstrated a strong effect on activated CD8^+^ TILs that was not be significantly enhanced by metformin. However, metformin increased infiltration of specific CD8^+^ TILs accompanied by IFN-γ production on the contralateral side compared to single-treated groups. Therefore, we concluded that metformin boost C-REV antitumor immunity by enhancing CD8^+^ TILs infiltration and activity.

Effector CD8^+^ T cells have a main role in antitumor immunity^47^. Here, our analysis revealed that our combination was able to induce CD8^+^ with high CD44^+^ and CD69^+^ in Pan02 tumors. CD8^+^ CD44^+^ are highly cytotoxic activated effector cells, which can induce antitumor response^34,48^. CD8^+^ CD69^+^ are effector T cell subset, which has a direct cytotoxic effect and it has an important role in acute immune response against tumors^34^. This fact was demonstrated when CD8^+^ CD69^+^ TILs expanded from patients with soft tissue sarcoma had more tumor-specific functional capacity due to more IFN-γ and granzyme B production^35^. In this study, we confirmed that combination therapy induces high infiltration of effector CD44^+^ and CD69^+^ CD8^+^ TILs, which may enhance antitumor activity. On the injected side, combination therapy shows high CD44^+^ or CD69^+^ CD8^+^ TILs infiltration compared to the control and metformin group, revealing the high impact of C-REV on these populations. However, on the contralateral side, the combination therapy showed significant infiltration against C-REV, which indicates the positive impact of metformin. Since CD44 and CD69 are reported as effector and activator markers for CD8^+^ TILs, we consider that CD44^+^ and CD69^+^ CD8^+^ TILs would have an antitumor effect against Pan02.

We previously reported that C-REV treatment enhanced new infiltration of CD8^+^ with low PD-1^-^ expression in the SCC-VII murine tumor model^16^. Since CD8^+^ PD-1^−^ TILs have high expansion ability compared to CD8^+^ PD-1^+^ TILs, we examined effector markers expression on it^49^. Our combination was able to induce CD8^+^ PD1^-^ with high CD44^+^ and CD69^+^ expression on both sides of tumor. The infiltration of these populations is more pronounced on the contralateral side, revealing antitumor activity (Fig. 3a–f). It was reported that CD44^+^ CD8^+^ PD1^-^ TILs are effector T cell subset as its lowly expressed lipid uptake receptor CD36. However, CD36 was enriched on exhausted CD44^+^ CD8^+^ PD-1^+^ TILs^50^. Interestingly, there was a positive correlation between CD44^+^ and CD69^+^ expression on CD8^+^ PD-1^−^ TILs on both sides of tumor. We concluded that metformin as well can enhance the C-REV effect on effector CD44^+^ and CD69^+^ CD8^+^ PD-1^−^ TILs infiltration. These populations may have an essential role in the antitumor response against Pan02 murine tumor model. Further studies are needed to highlight the role of CD44^+^ and CD69^+^ CD8^+^ PD-1^−^ in the antitumor immunity.

Ti-Treg cells are a main therapeutic target for cancer immunotherapy^51^. It was reported that HSV-1 armed IL-12 significantly increases the percentage of Ti-Treg cells in murine sarcoma model^52^. In contrast, HSV-1 encoding GM-CSF decreases frequency of Ti-Treg cells in tumor samples from melanoma patients^53^. In Pan02 murine model, our treatments did not affect the total percentage of Ti-Treg cells (Supplementary Fig. S3g). Thus, the tumor model and armed proteins are critical for the HSV-1 effect on Ti-Treg cells. CD103^+^ and KLRG1^+^ expression on T regulatory cells are indicators for terminally-differentiated cells (active effector T regulatory cells that recently responded to antigen)^54,55^. T regulatory cells expressing KLRG1^+^ CD103^+^ express both IL-10 and cytotoxic T lymphocyte antigen-4 (CTLA-4) inhibitory molecules^56,57^. In MethA tumor-bearing mice, metformin reduced terminally-differentiated T-reg cells infiltrated into the tumor^26^. However, there is no reports about the effect of HSV-1 oncolytic virus on terminally-differentiated Ti-Treg cells. Here, C-REV treatment does not impact the percentage of terminally-differentiated T-reg cells. However, metformin and combination treatments reduce the proportion of CD103^+^ KLRG-1^+^ from Ti-Treg population, which may maximize CD8^+^ TILs activity. Therefore, the negative impact of the combination on this population was mainly related to the systemic presence of metformin, which decreased the target population equally on both sides of tumor (Fig. 3g, h). Therefore, this treatment may improve the prognosis for cancer patients, especially the ones with low immunogenicity.

Our study also demonstrated the effect of combination treatment on innate DC immune response. Here, combination treatment increased DCs (CD11c^+^ MHC-II^+^) infiltration on both sides of tumor. Infiltrated DCs shows high MHC-I expression, indicating high cross-presentation ability^58^. cDC1 infinity in the TME is an indicator of immune-mediated rejection and successful immunotherapy^36^. It has the ability to release cytokines, which promote the recruitment of effector CD8 TILs^59^. Here, we confirmed that the cDC1 subset population was highly abundant after combination treatment especially on the injected side, which indicates the strong local antitumor effect. The high infiltration of cDC1 on both tumor sides is mainly contributed to the systemic presence of metformin (Fig. 4c, d).

XCR-1 is a chemokine receptor essential for cDC1 subset identification, which is responsible for cross-priming antitumor CD8^+^ T cells^38–40^. Here, we found that combination therapy induced a significant increase in XCR-1 expression on cDC1 on both sides of tumor, indicating the presence of functional DCs (Fig. 4e, f). Furthermore, we detected a high increase in cDC1 on TDLNs accompanied by high XCR-1 expression, which indicates DC activity (Fig. 4g, h). Thus, DCs migration into TDLNs enhances high effector CD8^+^ T cells infiltration into the contralateral side with high IFN-γ production. Therefore, the contralateral side of the combination group achieves a good antitumor effect (Fig. 1b). Based on our knowledge, this is the first paper to check the effect of oncolytic HSV-1 virus or metformin on XCR-1 expression on tumor and TDLNs cDC1. We conclude that metformin increases cDC1 infiltration that increases cross-presentation into infiltrated CD8^+^ TILs enhanced by C-REV, and thus, generates cytotoxic effector T cell. The positive impact of combination therapy on cDC1 infiltration was mainly related to the systemic administration of metformin. However, the mechanism that illustrates the metformin effect on cDC1 recruitment on both sides of tumor is still unknown. Further studies should elucidate the mechanism whereby metformin causes cDC1 recruitment and activation on TME.

C-REV treatment was injected intratumorally in phases I, and II clinical trials targeting melanoma, pancreatic, breast, head, and neck cancer^14^. Although intravenous injection of the oncolytic virus allows effective dissemination of the viruses into hidden tumors, the antitumor effect of intravenous injection of the oncolytic virus is limited such as in vaccinated or previously infected patients due to the possibility of the presence of neutralizing antibodies in their circulation^60^. It was reported that intravenous administration of Seprehvir; an oncolytic HSV-1 is well tolerated and showed an antitumor effect without any neurotoxicity. However, most of the patients were seroconverted 4 weeks following injection^61^. To overcome the limitation of humoral immune response against injected viruses, we previously showed that encapsulation of HSV-1 in liposomes did not attenuated by anti-HSV antibodies and it was effective to treat multiple liver metastases^62^.

Moreover, several strategies are developed to reduce viral clearance from circulation such as switching of viral serotype, blocking antibody binding to viral particles by covalent conjugation and polymer coating of the virus^63–65^. Moreover, the complexity of the TME may diminish the therapeutic efficacy of OVs. However, new combination strategies such as immunotherapy enhance the therapeutic efficacy of OVs^66^.

It is well known that genetic engineering of oncolytic viruses overcomes the limitation of OVs especially in solid tumors, via targeting of cancer cells or regulating the immune response. It has been reported that oncolytic adenovirus recombinant with the dual tumor suppressor genes ST13 and tumor necrosis factor-related apoptosis-inducing ligand (TRAIL) showed strong antitumor efficacy against PDAC^67^. Moreover, third-generation oncolytic HSV-1 G47∆, a triple mutant in the γ34.5, α47 and ICP6 genes, has a greater cytopathic effect and tumor-specific replication potential than G207 parent virus. Intratumoral administration of G47∆ showed a survival benefit and safety in residual or recurrent glioblastoma patients with high CD4^+^ and CD8^+^ TILs infiltration^68^. Therefore, G47∆ is the first oncolytic virus approved in Japan for treatment of patients with malignant glioma. On the other hand, some OVs are engineered to deliver immune regulatory factors such as Talimogene laherparepvec (T-VEC). T-VEC is a genetically modified HSV-1 generated by the deletion of ICP34.5 and ICP47 genes and insertion of human granulocyte–macrophage colony-stimulating factor (hGM-CSF) to promote DCs infiltration, which activates cytotoxic CD8^+^ T cells. T-Vec was the first OV to demonstrate clinical efficacy and safety in melanoma patients, and it is approved by the US Food and Drug Administration^69^. Since T-VEC and G47∆ modulates infiltrated immune cells in the tumor, T-VEC and G47∆ in combination with metformin may have good therapeutic potential.

Several studies have suggested that combining OVs or metformin with small molecule modulators for key signaling pathways could be a highly promising combination for cancer immunotherapy. These modulators can target signaling pathways for cancer cells and/or immune cells^70,71^. The PI3K/AKT/mTOR signaling pathway is one of the main targets of modulators due to its role in regulation of cell proliferation, metabolism and growth^72^. Several OVs combined with mTOR inhibitors such as Everolimus and rapamycin showed synergistic effect against several cancer cells^70^. This is likely supported by the fact that rapamycin significantly increases HSV replication and spread in tumor cells^73^. It is well known that metformin can inhibit cancer cell growth in vitro by inhibiting complex I of the ETC, resulting in decreased concentration of cellular ATPs, which in turn activates AMPK and inhibits mTOR^23^. However, in physiological condition, a plasma concentration of approximately 150 μM, is necessary to impair ATP synthesis in mice, which can be only achieved by oral administration of high doses of metformin (≥ 250 mg/kg)^74^. However, this dose is not realistic for patients taking standard clinical doses of metformin^75^. We showed that low concentrations of metformin did not affect the viability of tumor cells even in the combination with C-REV in vitro (Supplementary Fig. S1a). In addition, in our in vivo experiments, mice were orally administered with metformin (5 mg/ml), which is considered similar to the dose of metformin in diabetic patients^76^. Therefore, we speculate that our observed enhanced immune responses were not due to the mTOR pathway. However, it has been reported recently that a low dose of metformin can activate AMPK through direct binding to PEN2 with no effect on cellular AMP levels^75^. Activation of AMPK has been reported to be essential for glucose uptake by CD8^+^ T cells^77^. Therefore, we speculate that low dose of metformin is still able to activate AMPK, which promotes glucose uptake in CD8^+^ T cells. Indeed, it has been reported that metformin (5 mg/ml) reduces CD8^+^ TILs exhaustion and instead, enhances IFNγ production in CD8^+^ TILs through elevation of glucose transporter (Glut)-1 expression^25,45^.

In this study, we used a murine PDAC tumor model created with Pan02, and demonstrated the efficacy of C-REV and metformin combination and characterized antitumor immunity. However, the heterogeneity of human pancreatic cancer must be considered. Therefore, other murine PDAC tumor models should be used in the future studies related to the immunomodulatory effect of combination therapies.

In summary, we observed that the combination of C-REV with metformin may be a novel therapeutic combination. C-REV mainly enhances CD8^+^ TILs infiltration and function, which could be upregulated by metformin. Metformin also increases cDC1 infiltration as well as modulates suppressor terminally-differentiated Ti-Treg cells. Thus, this combination can enhance the antitumor effect. Currently, many OVs are undergoing clinical trials including C-REV in addition metformin is a highly prescribed medicine and millions of diabetic patients use this drug on a daily base. Our findings may provide new insights into the role of combination treatment in the modulation of immune-suppressive tumors.

## Methods

### Cell lines

Mouse Pan02 (RRID: CVCL_D627) and mouse SCC-VII (RRID: CVCL_V412) were kindly provided by Dr. Sho (Nara Medical University) and Dr. Masunaga (Kyoto University), respectively. African green monkey kidney cells (Vero cells; RRID: CVCL_0059) were obtained from the RIKEN cell bank (Tsukuba, Japan). All cell lines were cultured in Dulbecco’s modified eagle medium with high glucose (DMEM; Wako, Japan) and supplemented with 10% heat-inactivated fetal bovine serum (FBS; Biosera, France), 100 IU/ml penicillin, and 100 μg/ml streptomycin (Wako, Osaka, Japan) at 37 °C in a humidified atmosphere containing 5% CO2. All cell lines were tested by PCR for mycoplasma infection and cultured consecutively for at most 4 weeks. All experiments were performed with mycoplasma-free cells.

### Viruses

C-REV is an attenuated mutant clone derived from HSV-1 strain HF. The virus was propagated in Vero cells and stored in aliquots at − 80 °C. C-REV was diluted in PBS for in vivo and in vitro experiments. Viral titers were assayed in Vero cells and are expressed as plaque-forming units per milliliter (PFU/ml).

### Drugs

Metformin hydrochloride (1, 1-dimethylbiguanide hydrochloride) (138-18661) was purchased from Wako Pure Chemical Corporation, Osaka, Japan. Metformin is stored protected from light at 4 °C until further usage.

### Cell proliferation assay

Cell proliferation was determined using the 3-(4,5-dimethylthiazol-2-yl)-2,5-diphenyl tetrazolium bromide (MTT) dye reduction method. Pan02 were seeded, grown in DMEM, and incubated for 24 h at 37 °C with 5% CO2. After 24 h, infected with C-REV at several multiplicities of infection (MOIs) or treated with metformin then cells were cultured in high or low glucose medium, incubated at 37 °C with 5% CO2. The first day of treatment was defined as day 0, and cells were grown for 3 days. Viable cells were quantified by colorimetric MTT assays.

### Viral proliferation assay

Pan02 cells were placed on 24 well plates and incubated overnight. The following day, the cells were treated with C-REV at MOI of 1 and incubated with a low concentration of metformin (100 μM). Then, 24 and 48 h later, cells were scraped, and the supernatants were collected and subjected to three freeze–thaw cycles. The released virus particles were collected and serially diluted in DMEM without fetal bovine serum (FBS). Following a standard viral plaque assay^78^, Vero cells were infected with serial dilutions of viruses in 6-well plates for 1 h. The viral supernatant was removed, and 2% low-melting agarose was added. Cells were incubated at 37 °C for 5–7 days until the plaques could be counted.

### Tumor challenge and treatments in mice

Six- to seven-week-old female C57BL/6 mice were purchased from Japan SLC (Shizuoka, Japan). Mice were kept under constant temperature and humidity conditions and fed with a standard diet and water ad libitum. All mice were maintained under specific pathogen-free conditions. All experiments were reviewed and approved by the Animal Care University Committee following the Guidelines for Animal Experimentation at Nagoya University (Nos. 31322, 31323) (Nagoya, Japan). All methods were carried out in accordance with relevant guidelines and regulations. All methods are reported in accordance with ARRIVE guidelines.

A bilateral tumor model of Pan02 was used to evaluate antitumor effects. Tumors were cut into cubes (2 mm^3^). Pan02 tumors were inoculated into mice; one tumor cube was inoculated into each flank (right and left). When the average tumor size reached 100 mm^3^, treatments were then started on day 0. Mice were randomly divided into four groups (n = 4 mice/group) with an equal average tumor volume among the groups. C-REV (1 × 10^6^ PFU/100 μL PBS) was injected according to the experimental timeline on one side only (injected side). Metformin was continuously supplied in the drinking water (5 mg/ml) when C-REV treatment started. Clinical signs, body weight changes and tumor growth were monitored. Tumor volume was measured twice weekly until study termination. Tumor volume (V) was estimated using the equation V = L × W^2^/2, where L and W are tumor length and width, respectively.

### Tumor disaggregation and re-stimulation of tumor-infiltrating lymphocytes in vitro

Pan02 tumors were dissociated using a gentle MACS murine tumor dissociation kit (Miltenyi Biotec, Bergisch Gladbach, Germany) according to the manufacturer’s protocol. Briefly, tumors from treated mice were cut into 3 mm fragments and transferred into a C-tube (Miltenyi Biotec) with Enzyme A, Enzyme D, and Enzyme R in the kit. The samples were placed onto the GentleMACS dissociator according to the manufacturer’s instructions. After disaggregation, the cell suspension was filtered through a cell strainer (70 μm) and washed three times with PBS containing 0.1% BSA. Subsequently, tumor-infiltrating lymphocytes were re-stimulated as described^79^. Briefly, cells were labeled using a Miltenyi CD8α T cell enrichment kit (Miltenyi Biotec) and isolated using magnetic sorting according to the manufacturer’s protocols. Tissue culture plates were coated with 5 μg/mL anti-CD3 antibody (145-2C11; BioLegend, San Diego, CA) in PBS for 12 h, and excess antibody was aspirated before T cell addition. Cells were cultured for 48 h with 1 μg/mL anti-CD28 antibody before the addition of 2 μM monensin for 4 h for intracellular interferon γ (IFN-γ) staining.

### Preparation of single-cell suspensions of tumor-infiltrating lymphocytes and flow cytometry

Single-cell suspensions were obtained from mouse tumors after tumor disaggregation as described in the previous method. The cells were treated with anti-CD16/CD32 antibodies to block Fc receptors. Subsequently, the cells were stained with the following antibodies (BioLegend): Brilliant Violet 510-conjugated anti-CD45, FITC-conjugated anti-CD3, APC-Cy7-conjugated anti-CD8a, FITC-conjugated anti-CD8a, PerCP-conjugated anti-PD-1, APC- conjugated anti-CD44, Pacific Blue-conjugated anti-CD69, PE-conjugated anti-KLRG-1, PerCP-conjugated anti-CD25, APC- conjugated anti-CD103, APC-Cy7-conjugated anti-CD4, Pacific Blue-conjugated anti-FOXP3, PerCP-conjugated anti-MHC-II, APC-Cy7-conjugated anti-CD11c, Pacific Blue-conjugated anti-CD11b, PE-conjugated anti-XCR-1. The cells were stained for 30 min at 4 °C in the dark. For intracellular staining, cells were fixed using 4% Paraformaldehyde Phosphate Buffer Solution (Wako, Osaka, Japan) and permeabilized using 0.5% Polyoxyethylene (10) Octylphenyl Ether (Wako, Osaka, Japan) then stained with antibody for 30 min at 4 °C in the dark. After extensive washing with FACS buffer, the cells were subjected to flow cytometry Canto II flow cytometer (BD Biosciences, San Jose, CA). Data were analyzed using FlowJo software (BD Biosciences, version 10.6).

### Statistical analysis

Statistical comparisons were performed using the Prism software, version 9.3.1 (GraphPad Software). Statistical significance between two groups was analyzed using student’s *t-*test. One-way ANOVA with a post-hoc Tukey’s test was performed to analyze flow cytometry data. Two-way ANOVA with Tukey’s post-test was used for experiments involving the analysis of multiple time points. p-values < 0.05 were considered to be statistically significant.

## Supplementary Information

Supplementary Information.

## Data Availability

All data generated or analyzed during this study are included in this published article and its supplementary information files.
