# Supplementary material for: Metformin enhances the antitumor activity of oncolytic herpes simplex virus HF10 (canerpaturev) in a pancreatic cell cancer subcutaneous model
Source: Sci Rep. 2022 Dec 13;12:21570. doi: 10.1038/s41598-022-25065-w (PMC9747797; doi:10.1038/s41598-022-25065-w)
Supplement: Supplementary file 1 — Supplementary Information. [file 41598_2022_25065_MOESM1_ESM.pdf]

Supplementary Material

Metformin enhances the antitumor activity of oncolytic herpes simplex virus HF10 (canerpaturev) in a pancreatic cell cancer subcutaneous model

Mohamed Abdelmoneim, Ibrahim R. Eissa, Mona Al Hussein Mostafa Aboalela, Yoshinori Naoe, Shigeru Matsumura, Patricia A. Sibal, Itzel Bustos-Villalobos, Maki Tanaka, Yasuhiro Kodera & Hideki Kasuya

Supplementary Figure 1. Metformin does not enhance cell cytotoxicity induced by C-REV *in vitro*.

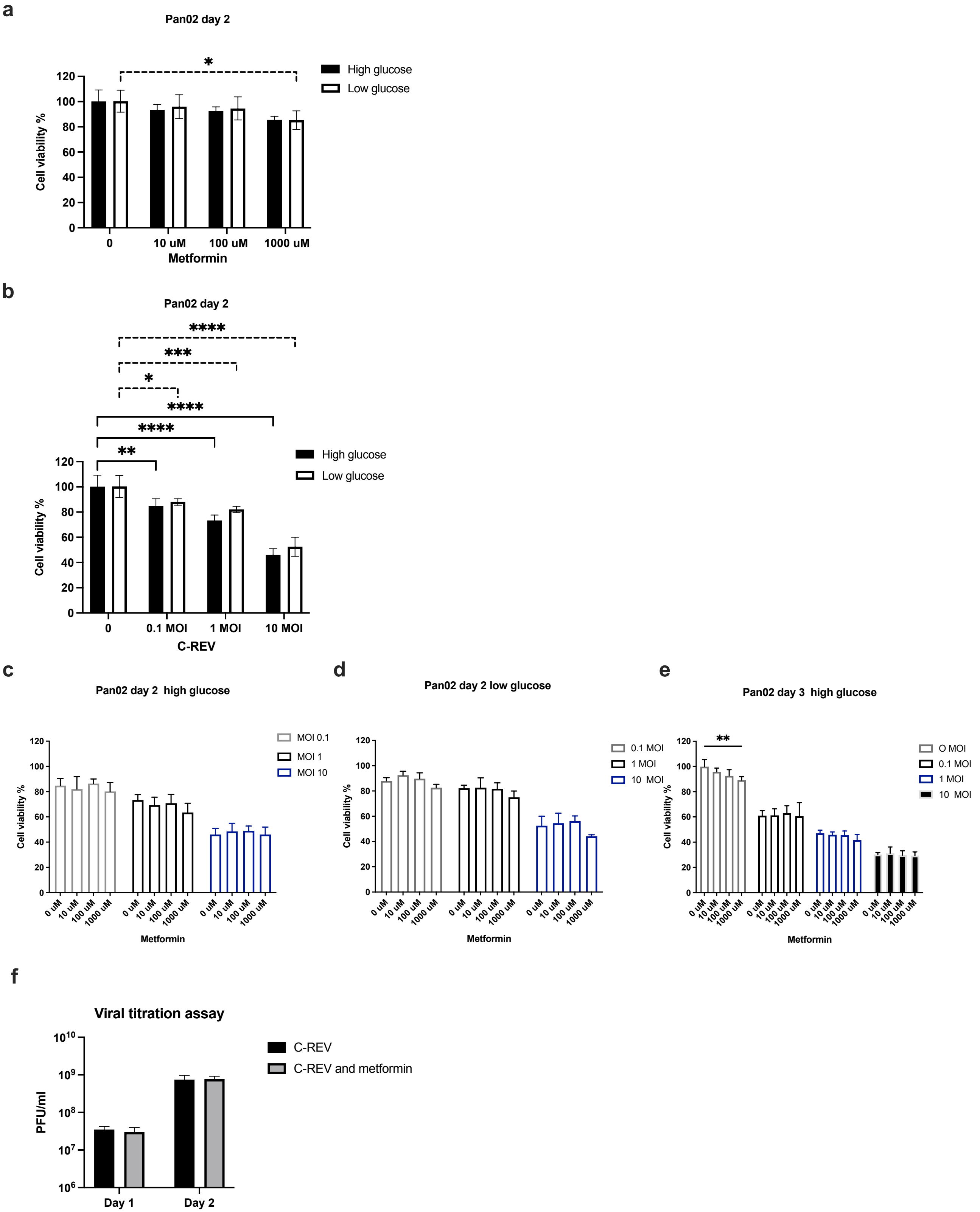

Supplementary Figure 1. Metformin does not enhance cell cytotoxicity induced by C-REV *in vitro*. **(a)** Cytotoxicity of Pan02 after treatment with different concentrations of metformin (0, 10 uM, 100 uM, 1 mM) in medium containing high and low glucose (25 mM and 5.5 mM) for 2 days was determined by MTT assay. **(b)** Cytotoxicity of Pan02 after treatment with different MOI (0, 0.1, 1 and 10 MOI) of C-REV in medium containing high and low glucose for 2 days was determined by MTT assay. **(c, d)** Cytotoxicity of Pan02 after treatment with metformin and infected with several MOI of C-REV in medium containing high (c) and low (d) glucose for 2 days was determined by MTT assay.

(e) Cytotoxicity of Pan02 after treatment with metformin and infected with several MOI of C-REV in medium containing high glucose for 3 days was determined by MTT assay. Data are presented as mean ± SD (n=6). Each experiment was coducted at least three times, yielding similar results. Two-way ANOVA followed by Tukey's multiple comparisons tests was performed. (f) *In vitro* replication of C-REV (MOI 1) over a 2-day peroid, co-incubated with 100 uM of metformin, as assessed by viral titer. Data are presented as mean ± SD (n=3). * $p < 0.05$, ** $p < 0.01$, *** $p < 0.001$, **** $p < 0.0001$.

Supplementary Figure 2. Combination therapy does not increase IFN-γ production from $CD8^{+}$ $CD3^{+}$ TILs at day 3.

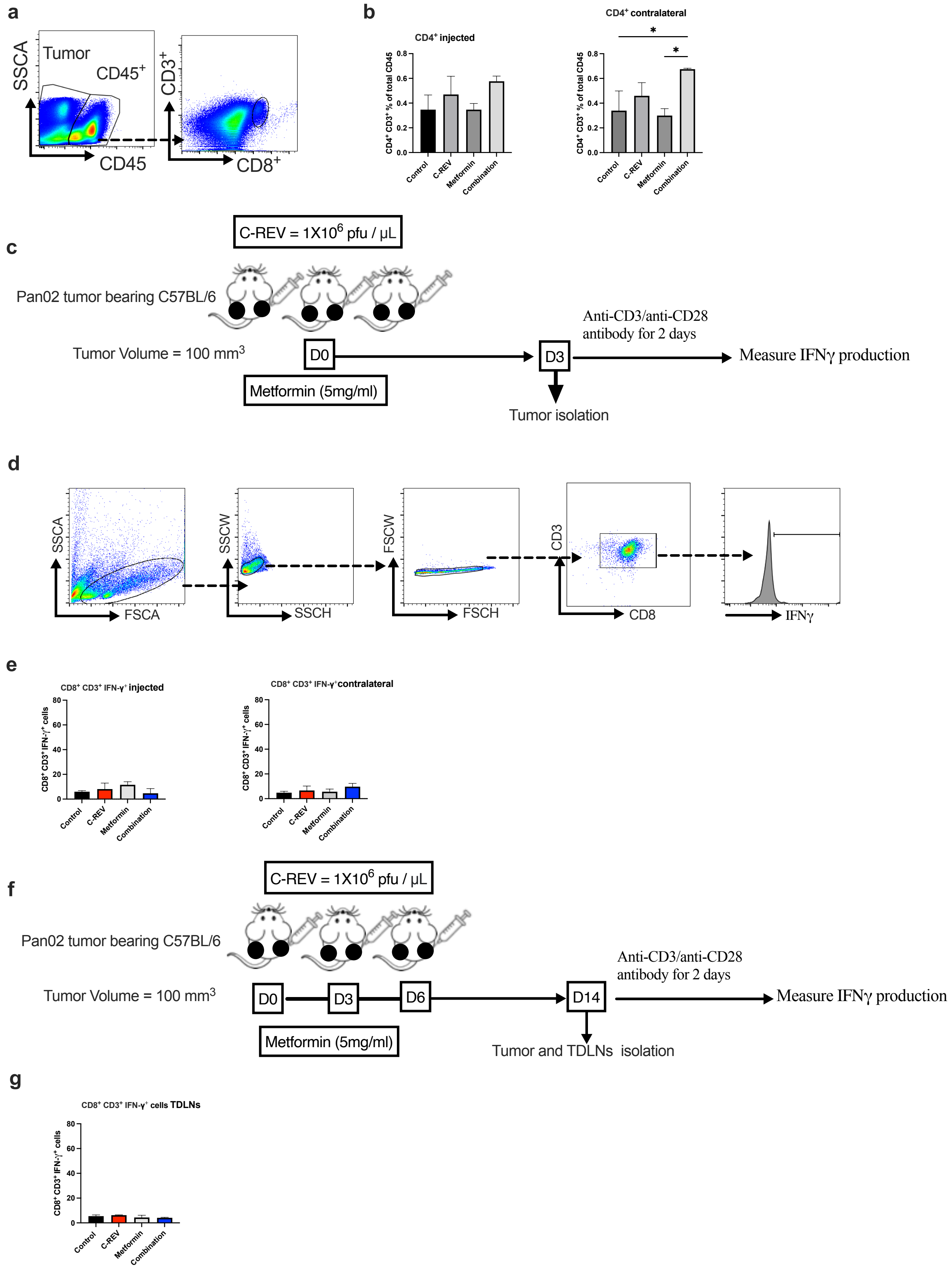

Supplementary Figure 2. Combination therapy does not increase IFN-γ production from $CD8^{+}$ $CD3^{+}$ TILs at day 3. (a) Gating strategy for detection of $CD8^{+}$ $CD3^{+}$ and $CD4^{+}$ $CD3^{+}$TILs. **(b)** Bar graphs show the percentage $CD4^{+}$ $CD3^{+}$ cells on the injected and contralateral sides. **(c)** A scheme shows schedule of C-REV and metformin treatment in C57BL/6 tumor-bearing mice for 3 days. **(d)** Gating strategy for detection of IFN-γ production from $CD8^{+}$ $CD3^{+}$ TILs. **(e)** Bar graphs show the percentage of IFN-γ production from $CD8^{+}$ $CD3^{+}$ cells on the injected and contralateral sides. **(f)** A scheme shows the schedule of C-REV and metformin treatment in C57BL/6 tumor-bearing mice for 14 days. **(g)** A bar graph shows the percentage of IFN-γ production from $CD8^{+}$ $CD3^{+}$ cells in TDLNs. Data are presented as mean ± SD (n=3 mice). One-way ANOVA with a post-hoc Tukey's tests was performed.

Supplementary Figure 3. Effect of combination therapy on T cell population in tumors and TDLNs.

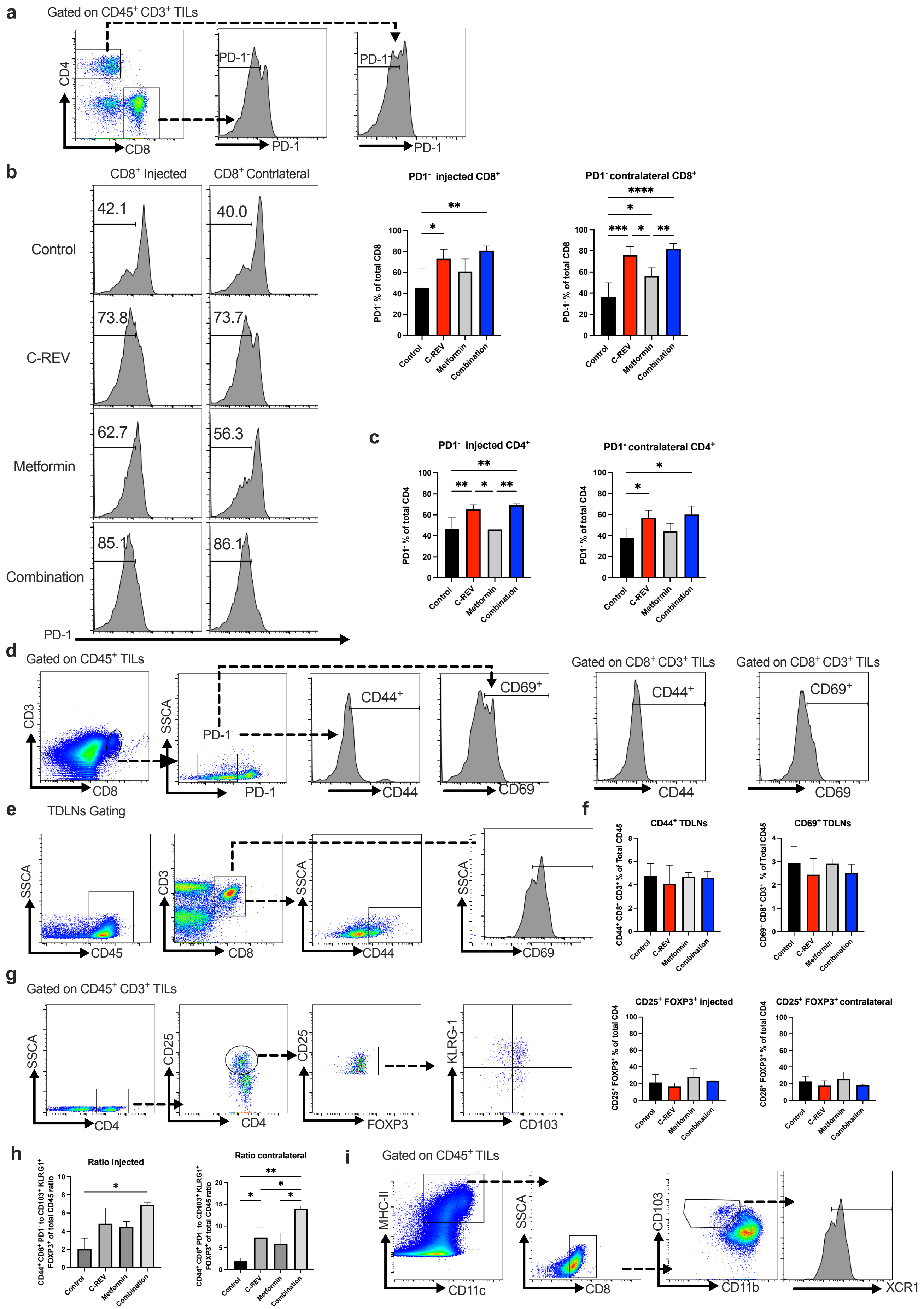

Supplementary Figure 3. Effect of combination therapy on T cell population in tumors and TDLNs. Mice were inoculated with Pan02 tumors as in Fig. 1A, tumors, and TDLNs were collected as previously shown. **(a)** Gating strategy for detection of PD-1^- expression on $CD8^+$ and $CD4^+$ TILs. **(b)** Representative histograms show PD-1^- expression on $CD8^+$ TILs cells on the injected and contralateral sides and bar graphs show percentage of PD-1^- expression on $CD8^+$ TILs cells on the injected and contralateral sides. **(c)** Bar graphs show the percentage of PD-1^- expression on $CD4^+$ TILs cells on the injected and contralateral sides. **(d)** Gating strategy for detection of $CD44^+$ and $CD69^+$ expression on $CD8^+$ $CD3^+$ and PD-1^- $CD8^+$ TILs. **(e)** Gating strategy for detection of $CD44^+$ and $CD69^+$ expression on $CD8^+$ $CD3^+$ in TDLNs. **(f)** Bar graphs show the percentage of $CD44^+$ and $CD69^+$ on $CD8^+$ $CD3^+$ cells in TDLNs. Data are presented as mean ± SD (n=3 mice). **(g)** Gating strategy for detection of $CD103^+$ KLRG-1^+ expression on $CD4^+$ $CD25^+$ $FOXP3^+$and bar graphs show the percentage of $CD4^+$ $CD25^+$ $FOXP3^+$ tumor-infiltrating T-reg cells on the injected and contralateral sides. (h) Bar graphs shows the ratio between $CD44^+$ $CD8^+$ PD-1^- population and terminally differentiated $CD103^+$ KLRG-1^+ T-reg cells on the injected and contralateral sides **(i)** Gating strategy for detection of XCR-1 expression on cDC1 in tumor . Data are presented as mean ± SD (n=4 mice). This experiment was conducted at least two times . One-way ANOVA with a post-hoc Tukey's tests was performed.* $p < 0.05$, ** $p < 0.01$, *** $p < 0.001$, **** $p < 0.0001$.
